# Validation of the Emergency Department-Paediatric Early Warning Score (ED-PEWS) for use in low- and middle-income countries: A multicentre observational study

**DOI:** 10.1371/journal.pgph.0002716

**Published:** 2024-03-21

**Authors:** Naomi Kemps, Natanael Holband, Navin P. Boeddha, Abdoulie Faal, Amadu E. Juliana, Godfrey A. Kavishe, Kristina Keitel, Kevin H. van ‘t Kruys, Elizabeth V. Ledger, Henriëtte A. Moll, Andrew M. Prentice, Fatou Secka, Rainer Tan, Effua Usuf, Stefan A. Unger, Joany M. Zachariasse, on behalf of VITaLs (VItal signs and Triage in Low- and middle income countries) study group

**Affiliations:** 1 Department of General Paediatrics, Erasmus MC- Sophia Children’s Hospital, University Medical Centre Rotterdam, Rotterdam, The Netherlands; 2 Department of Paediatrics, Academic Hospital Paramaribo, Paramaribo, Suriname; 3 Applications Development & e-Health Department, Medical Research Council Unit The Gambia at the London School of Hygiene and Tropical Medicine, Fajara, The Gambia; 4 National Institute of Medical Research–Mbeya Medical Research Centre, Mbeya, Tanzania; 5 Division of Paediatric Emergency Medicine, Department of Paediatrics, Inselspital, Bern University Hospital, Bern, Switzerland; 6 Swiss Tropical and Public Health Institute (SwissTPH), University of Basel, Basel, Switzerland; 7 Department of Paediatrics, Bristol Royal Hospital for Children, Bristol, The United Kingdom; 8 Nutrition and Planetary Health Theme, Medical Research Council Unit The Gambia at the London School of Hygiene & Tropical Medicine, Banjul, The Gambia; 9 Medical Research Council Unit The Gambia at the London School of Hygiene and Tropical Medicine, Fajara, The Gambia; 10 Center for Primary Care and Public Health (Unisanté), University of Lausanne, Lausanne, Switzerland; 11 Department of Child Life and Health, University of Edinburgh, Edinburgh, The United Kingdom; T D Medical College, INDIA

## Abstract

Early recognition of children at risk of serious illness is essential in preventing morbidity and mortality, particularly in low- and middle-income countries (LMICs). This study aimed to validate the Emergency Department-Paediatric Early Warning Score (ED-PEWS) for use in acute care settings in LMICs. This observational study is based on previously collected clinical data from consecutive children attending four diverse settings in LMICs. Inclusion criteria and study periods (2010–2021) varied. We simulated the ED-PEWS, consisting of patient age, consciousness, work of breathing, respiratory rate, oxygen saturation, heart rate, and capillary refill time, based on the first available parameters. Discrimination was assessed by the area under the curve (AUC), sensitivity and specificity (previously defined cut-offs < 6 and ≥ 15). The outcome measure was for each setting a composite marker of high urgency. 41,917 visits from Gambia rural, 501 visits from Gambia urban, 2,608 visits from Suriname, and 1,682 visits from Tanzania were included. The proportion of high urgency was variable (range 4.6% to 24.9%). Performance ranged from AUC 0.80 (95%CI 0.70–0.89) in Gambia urban to 0.62 (95%CI 0.55–0.67) in Tanzania. The low-urgency cut-off showed a high sensitivity in all settings ranging from 0.83 (95%CI 0.81–0.84) to 1.00 (95%CI 0.97–1.00). The high-urgency cut-off showed a specificity ranging from 0.71 (95%CI 0.66–0.75) to 0.97 (95%CI 0.97–0.97). The ED-PEWS has a moderate to good performance for the recognition of high urgency children in these LMIC settings. The performance appears to have potential in improving the identification of high urgency children in LMICs.

## Introduction

In high- and low-income countries millions of patients seek emergency care yearly, approximately a quarter of whom are children. In these acute care settings, recognizing the child with serious illness or at risk of deterioration is a major clinical challenge [1]. In hospitals in low-income countries about 50% of deaths of children occur in the first 24 hours after admission [2–4]. Therefore, effective and adequate prioritisation of children based on their urgency is crucial to ensure that the most severely ill children are identified early and accurately, and resources are efficiently allocated. Better prioritisation of high urgent children starts with accurate triage upon first presentation.

Several triage systems have been developed specifically for use in low- and middle-income countries (LMICs), such as the Emergency Triage Assessment and Treatment (ETAT) [5]. In contrast to conventional triage systems, Paediatric Early Warning Scores (PEWS) are simple scoring systems solely based on physiological parameters, aimed to assess the severity of illness and risk of clinical deterioration. PEWS are quick, easily applicable at the bedside, and consist of objective parameters, which is an advantage in settings where patients speak different local languages.

Research on the use of PEWS in LMICs is scarce and there is no consensus on which PEWS has the best performance (S1 File). A promising tool is the recently developed Emergency Department-Paediatric Early Warning Score (ED-PEWS) [6]. This is the first score fully based on statistical modelling and specifically developed for acute care settings. In the original study, the ED-PEWS showed a good performance for identifying both high and low urgency patients in a large European cohort including more than 100,000 paediatric visits. However, performance of the ED-PEWS in LMICs is unknown. Therefore, the aim of this study is to validate the ED-PEWS in different low- and middle income settings.

## Methods

### Study design

This study is an observational study, based on four databases with previously collected clinical data from The Gambia (rural and urban), Suriname and Tanzania. All databases have previously been created for research purposes [7–12]. The data were collected during routine clinical care or during clinical care as part of a research setting. The local medical ethical committees approved of the data collection: Gambia Government/MRCG Joint Ethics Committee, Project ID: 24006 (Gambia rural), and 22470 (Gambia urban); Commissie Mensgebonden Wetenschappelijk Onderzoek Ministerie van Volksgezondheid Paramaribo, 01 februari 2021; and Ifakara Health Institute (IHI/IRB/No: 12–2014). The requirement for informed consent was waived in The Gambia rural and Suriname. A declaration of exemption (“verklaring niet-WMO plichtig”) from the Erasmus MC Ethics committee has been issued. We followed the TRIPOD (Transparent Reporting of a multivariable prediction model for Individual Prognosis Or Diagnosis) statement for reporting [13].

### Study sites and population

We recruited patient cohorts that consisted of consecutive children attending primary and emergency care settings in LMICs and had data collected on physiological parameters and markers of severity [14]. This resulted in data from four cohorts from heterogeneous acute care settings in three different countries (Table 1). In short data was collected from 1) the MRC Keneba Fieldstation, a primary healthcare clinic in a rural region Kiang West in The Gambia (hereafter: Gambia rural); 2) MRC Fajara and Kanifing general hospital, two urban tertiary care clinics in The Gambia (Gambia urban); 3) Academic hospital Paramaribo, a tertiary care hospital in an urban area in Suriname (Suriname); and 4) three secondary care district hospitals and six primary health care centres in Tanzania (Tanzania). The inclusion criteria of these databases were defined by the original study for which the data was collected. The most important inclusion criteria were fever (Tanzania and Gambia urban) and age under 60 months (Tanzania). All settings were open access and there was no referral necessary by another physician. All duplicates, children with missing data on age, and in Gambia rural children with mothers under fifteen years of age were excluded. Furthermore, we did not include routine visits unrelated to emergency care, e.g. immunisations and follow-up visits. All databases were checked for quality and implausible values. The quality of the data was assessed frequency tables of the variables, and specifically by assessing outliers in each age category. Additionally, discrepancies in the data were discussed with the local research teams. Data checks and interpretation took place in close collaboration with the local research teams. For each of the settings we scheduled a minimum of two online meetings to discuss the database and findings.

**Table 1:** Characteristics of the study settings.

	Gambia Rural	Gambia Urban	Suriname	Tanzania
Study setting	MRC at London School of Hygiene & Tropical Medicine Keneba Fieldstation, Kiang West	MRC at London School of Hygiene & Tropical Medicine clinical service department Fajara, and Kanifing General hospital	Academic hospital Paramaribo	Three district hospitals and six health centres in Dar es Salaam
Setting type	Primary health care clinic in a rural area	Two tertiary health care clinics in urban areas	Tertiary care clinic in urban area	Three secondary care district hospitals and six primary health care centres
Country	The Gambia	The Gambia	Suriname	Tanzania
Income level [14]	Low income	Low income	Upper-middle income	Lower-middle income
Inclusion criteria	All children < 15 years of age, resident in the Kiang West region	Children <18 years of age with fever (temperature measured ≥ 38.0°C or history of fever in the preceding 72 hours) and an indication for a blood draw	All children <16 years of age	Children 2–59 months of age with fever (history of fever in the preceding 7 days and axillary temperature ≥37.5°C), randomized to ePOCT arm of trial
Study period	January 2010 to December 2014	December 2016 to October 2018	February 2021 to August 2021	December 2014 to February 2016
Data collection	Previously collected from the Gambia Rural Keneba Electronic Medical Records System (KEMReS) [7, 8, 10]	Previously collected for the Biomarker Validation in the Emergency Department (BIVA-ED) study as part of the PERFORM study [9, 11]	Prospective data-collection by trained medical students as part of the Pediatric Early Detection of Severe infections in Suriname (PEDSS) study	Previously collected for the ePOCT trial [12]
Number of included visits	41,917	501	2,608	1,596

MRC = Medical Research Council

### Vital signs and ED-PEWS

We simulated the ED-PEWS using the available vital signs data. In all settings, vital signs were taken on presentation by trained personnel according to local practices (S2 File). Only the first set of clinical observations for each child were included. The ED-PEWS was retrospectively calculated using the following vital signs and physiological parameters: age, heart rate, respiratory rate, oxygen saturation, capillary refill time, consciousness, and work of breathing. To each vital sign a score was assigned and all scores were added up to reach the total score. This total score can range from 0–68 points (S3 File) [6]. Capillary refill time was not available in the Tanzanian data. If a variable was not available or missing, we scored the variable as normal. Due to the fact that all variables had less than 5% missing data a complete records analysis was considered acceptable for the logistic regression analysis and furthermore this is a representation of how the score would be used in clinical practice because an unavailable item would not contribute to the total score [15].

In addition to the vital signs, we collected data on existing triage methods and whether staff considered a child as ill appearing. In all settings except for Tanzania, patients were routinely triaged using a conventional triage system. In Gambia rural, triage was performed using the 3-level Emergency Triage Assessment and Treatment (ETAT) [5]. In Suriname the 5-level system the Emergency Severity Index (ESI), and in Gambia urban, the 5-level Integrated Management of Childhood Illness (IMCI) classifications were used [5, 16]. Both the ESI and the ETAT make use of vital parameters or derivatives of vital parameters in their respective triage systems. Ill appearance was assessed by the attending healthcare workers based on their own judgement.

### Outcome measures

The outcome measure representing high urgent children was defined for each setting separately. We used the original outcome measure from the ED-PEWS derivation study as starting point (S4 File), and together with the local research teams, adjusted the different items to the local situation, taking into account the available data. In all settings, a marker of a higher level of care, for example intensive care unit (ICU) admission or referral to secondary care, was selected if available. Furthermore, admission, and in some settings intravenous (IV) medication were part of the outcome measure. Additional available markers for high urgency in Suriname were oxygen administration, inhalation medication and immediate lifesaving interventions (Table 2) [17, 18]. All variables had been collected prior to start of the study, and thus there was no need for blind assessment of predictor and outcome variables.

**Table 2:** High urgency outcome measures in different study sites.

	Gambia Rural	Gambia Urban	Suriname	Tanzania
Outcome measures	• Death on day of presentation • Admission • Referral • IV medication	• Death on day of presentation • Admission	• Death on day of presentation • ICU admission • Immediate lifesaving interventions • Admission • IV medication • Inhalation medication • Oxygen administration	• Death on day of presentation • Admission • Referral

IV = intravenous, ICU = intensive care unit

### Statistical analysis

The data were statistically analysed with SPSS version 25. In a descriptive analysis, the baseline characteristics of the presenting children were explored. Subsequently, univariable and multivariable logistic regression models were used to calculate the association between the individual predictors of the ED-PEWS and the outcome, adjusted for age, sex and other vital parameters. Temperature was not included in the analyses for Gambia urban and Tanzania because fever was an inclusion criterion for these databases. The analyses from Gambia rural were also adjusted for free access to transportation to the clinic, because this was an important confounder in this population in previous research [8, 19]. Free access to transportation was defined as children living in Keneba, Manduar or Kantong Kunda, who received free weekly transport to the clinic during the study period.

Discrimination of the ED-PEWS was quantified by the area under the curve (AUC). We were unable to determine measures of calibration (the agreement between observed outcomes and predictions), because our outcome measure differed from the original derivation study. To assess the ED-PEWS’ diagnostic accuracy, we calculated sensitivity, specificity, positive predictive value (PPV), negative predictive value (NPV), positive likelihood ratio and negative likelihood ratio. In the original validation of the ED-PEWS in European data, the cut-off for low-urgency was defined as score <6, and a score ≥15 was defined as the cut-off for high urgency [6]. These pre-determined cut-offs were used for the diagnostic accuracy calculations. Furthermore, we explored a range of potentially relevant cut-off values for high and low urgency. We defined the optimal cut-off as sensitivity > 0.9 for low-urgency and a specificity of > 0.9 for high urgency. Subgroup-analyses were performed to assess the performance of the ED-PEWS in children under five years, and in children with fever, defined as a temperature above 38.0°C. The results from the ED-PEWS were compared to Egdell’s Paediatric Advanced Warning score (PAWS) (S5 File) and the existing triage system in each setting [20]. The Egdell-PAWS is one of the few PEWS specifically designed for use in acute care settings, although it was developed in a high-income country. The PAWS was retrospectively calculated based on the following variables: age-adjusted respiratory rate, age-adjusted heart rate, saturation, temperature, consciousness, capillary refill time and increased work of breathing. We had to adjust the levels of consciousness based on the variables available in the database.

The study size was based on a convenience sample of four databases from LMICs. Due to the fact that all the data was collected prior to the study, we only assessed if we had enough cases to confidently draw any conclusions about the data. The most important considerations were the amount of high urgent children. For external validation, simulation studies have suggested a minimum of 100 events, which would mean high-urgent children, and 100 non-events or low-urgent children, to obtain reliable estimates of model performance [21, 22].

### Role of the funding source

The funder of the study had no role in study design, data collection, data analysis, data interpretation or writing of the report.

## Results

### Baseline characteristics

Data were available from 54,192 visits to one of the four different study sites. A total of 7,537 visits (14%) were excluded, the majority because they were defined as routine visits not related to emergency care (n = 7,429). After application of the exclusion criteria, 46,652 visits remained: 41,917 visits from Gambia rural, 501 visits from Gambia urban, 2,608 visits from Suriname and 1,596 visits from Tanzania (Table 3 and S6 File). The median patient age ranged from 1.1 to 4.2 years and the majority of children were boys (range 51.6% to 56.4%). Following the initial visit, the percentage of children admitted to hospital including the ICU ranged from 3.4% to 14.3%. The proportion of children with the high urgency outcome as described in Table 2, ranged from 4.6% (Gambia Urban) to 24.8% (Suriname). In all centres, missing rates of physiological parameters were low (S7 and S8 Files).

**Table 3:** Baseline characteristics of the study population.

	Gambia Rural (n = 41 917)	Gambia Urban (n = 501)	Suriname (n = 2 608)	Tanzania (n = 1 596)
Age				
Median (IQR)	4.2 (1.5–9.7)	3.1 (1.6–5.4)	4.0 (1.7–8.8)	1.1 (0.7–1.9)*
Sex, n (%)				
Male	21,649 (51.6)	264 (52.7)	1,479 (56.7)	891 (55.8)
Female	20,268 (48.4)	237 (47.3)	1,126 (43.2)	705 (44.2)
Unknown	··	··	3 (0.1)	··
Ill Appearance, n (%)				
Yes	1,790 (4.2)	100 (20.0)	637 (24.4)	··
No	40,097 (95.7)	400 (79.8)	1,819 (69.7)	··
Unknown	30 (0.1)	1 (0.2)	152 (5.8)	··
Triage label according to existing triage, n (%)				
Immediate	118 (0.3)	1 (0.2)	88 (3.4)	··
Very urgent	··	1 (0.2)	1,061 (40.7)	··
Urgent	2,987 (7.1)	23 (4.6)	643 (24.7)	··
Standard	36,681 (87.5)	475 (94.8)	687 (26.3)	··
Non-urgent	··	1 (0.2)	29 (1.1)	··
Unknown	2,131 (5.1)	··	100(3.8)	··
Action after visit, n (%)				
Home	39,204 (93.5)	479 (95.6)	2,157 (82.7)	1,489 (93.3)
Admission to ward	1,427 (3.4)	22 (4.4)	332 (12.7)	46 (2.9)
Admission to PICU/NICU	··	··	42 (1.6)	··
Referral to higher level of care	327 (0.8)	··	··	60 (3.8)
Other	315 (0.8)	··	74 (2.8)	··
Unknown	664 (1.5)	0 (0)	3 (0.1)	··
Death on day of presentation, n (%)				
Yes	4 (0.1)	1 (0.2)	5 (0.2)	0 (0.0)
No	41,913 (99.9)	500 (99.8)	2,603 (99.8)	1,596 (100.0)
Intravenous Medication, n (%)				
Yes	800 (1.9)	20 (4.0)	134 (5.1)	··
No	41,117 (98.1)	481 (96.0)	2,474 (94.9)	··
Unknown	··	··	··	··
High urgency outcome measure, n (%)				
Yes	2,054 (4.9)	23 (4.6)	648 (24.8)	106 (6.6)
No	39,863 (95.1)	478 (95.4)	1,960 (75.2)	1,490 (93.4)

* Inclusion criteria include age 2 to 59 months

All variables, except capillary refill time in Gambia rural, and work of breathing in Tanzania, were significantly associated with the outcome measures in the multivariable analysis (S9 File). The strongest predictors were consciousness with an adjusted OR ranging from 1.8 (95%CI 1.3–2.5) to 5.5 (95%CI 3.3–9.3) and work of breathing with an adjusted OR ranging from 2.6 (95%CI 2.0–3.3) to 9.6 (95%CI 6.4–14.3).

### Performance of the ED-PEWS

Performance of the ED-PEWS was highly variable in the different settings. The AUC was highest in Gambia urban (0.80 (95%CI 0.70–0.89)), followed by Suriname (0.78 (95%CI 0.76–0.81)) and lowest in Gambia rural (0.67 (95%CI 0.65–0.68)) and Tanzania (0.62 (95%CI 0.55–0.67)) (Table 4). When looking at the different subgroups, performance was better in the children under five years compared with older children. There was no consistent trend in performance in the subgroup of children with fever. In all settings, discrimination of the ED-PEWS was better than the Egdell-PAWS and the existing local triage methods, except for the existing triage in Gambia urban (Table 4).

**Table 4:** Performance of the Emergency Department-Paediatric Early Warning Score.

	Gambia Rural	Gambia Urban	Suriname	Tanzania
	AUC (95%CI)	AUC (95%CI)	AUC (95%CI)	AUC (95%CI)
ED-PEWS	0.67 (0.65–0.68)	0.80 (0.70–0.89)	0.78 (0.76–0.81)	0.62 (0.55–0.67)
Subgroups				
< 5 years	0.68 (0.67–0.69)	0.84 (0.72–0.96)	0.80 (0.77–0.83)	··
≥ 5 years	0.62 (0.59–0.64)	0.72 (0.57–0.97)	0.76 (0.72–0.79)	··
Fever	0.72 (0.70–0.74)	··	0.72 (0.64–0.80)	··
No fever	0.61 (0.60–0.62)	··	0.78 (0.75–0.80)	··
Comparison to other scores				
Existing triage*	0.58 (0.57–0.60)	0.88 (0.78–0.99)	0.71 (0.68–0.73)	··
Egdell-PAWS	0.64 (0.63–0.66)	0.70 (0.59–0.83)	0.75 (0.72–0.78)	0.58 (0.52–0.65)

AUC = Area under the curve

* 3-level Emergency Triage Assessment and Treatment (ETAT, Gambia rural), 5-level Emergency Severity Index (ESI, Suriname), and 5-level Integrated Management of Childhood Illness (IMCI, Gambia urban)

The earlier defined low-urgency cut-off point of < 6 showed a high sensitivity in all settings ranging from 0.83 (95%CI 0.81–0.84) to 1.00 (95%CI 0.97–1.00) and identified 2.7% to 33.0% of all visits as low urgent (Table 5). The earlier defined high-urgency cut-off point of ≥15 showed a specificity ranging from 0.71 (95%CI 0.66–0.75) to 0.97 (95%CI 0.97–0.97) and classified 3.4% to 30.9% as high urgent. The optimal cut-off points were only explored in Gambia rural, Gambia urban and Suriname, because the restricted age group in Tanzania made it impossible to define a representative cut-off (S10 File). The earlier defined cut-off points were optimal for Suriname and a reasonable choice for Gambia rural and Gambia urban. A better cut-off point in Gambia rural would be ≥ 10 for high urgency, classifying 10.1% as high urgent, with a specificity of 0.91 (95%CI 0.91–0.91), and in Gambia urban ≥ 20 for high urgency, classifying 8.4% as high urgent, with a specificity of 0.93 (95%CI 0.91–0.95) and < 10 for low urgency, classifying 28.5% as low urgent, with a sensitivity of 0.96 (95%CI 0.78–1.00).

**Table 5:** Diagnostic accuracy of the Emergency Department-Paediatric Early Warning Score.

Cut-off	Patients classified (%)	Sensitivity (95%CI)	Specificity (95%CI)	PPV (95%CI)	NPV (95%CI)	Positive LR (95%CI)	Negative LR (95%CI)
Gambia Rural							
**<6**	33.0	0.83 (0.81–0.84)	0.34 (0.33–0.34)	0.06 (0.06–0.06)	0.97 (0.97–0.98)	1.3 (1.2–1.3)	0.51 (0.46–0.56)
**≥15**	3.4	0.16 (0.15–0.18)	0.97 (0.97–0.97)	0.23 (0.21–0.26)	0.96 (0.96–0.96)	6.0 (5.3–6.7)	0.86 (0.84–0.88)
Gambia Urban							
**<6**	5.4	1.00 (0.85–1.00)	0.06 (0.04–0.08)	0.05 (0.05–0.05)	1.00 (1.00–1.00)	1.1 (1.0–1.1)	0.00 (0.00–0.00)
**≥15**	30.9	0.65 (0.43–0.84)	0.71 (0.66–0.75)	0.10 (0.07–0.13)	0.98 (0.96–0.99)	2.2 (1.6–3.1)	0.49 (0.28–0.86)
Suriname							
**<6**	32.5	0.87 (0.84–0.89)	0.39 (0.37–0.41)	0.32 (0.31–0.33)	0.90 (0.88–0.92)	1.4 (1.4–1.5)	0.33 (0.27–0.41)
**≥15**	15.7	0.47 (0.43–0.51)	0.95 (0.94–0.96)	0.75 (0.71–0.79)	0.84 (0.84–0.85)	9.1 (7.4–11.1)	0.56 (0.52–0.60)
Tanzania							
**<6**	2.7	1.00 (0.97–1.00)	0.03 (0.02–0.04)	0.07 (0.07–0.07)	1.00 (1.00–1.00)	1.0 (1.0–1.0)	0.00 (0.00–0.00)
**≥15**	26.6	0.42 (0.32–0.51)	0.75 (0.72–0.77)	0.10 (0.08–0.13)	0.95 (0.94–0.95)	1.6 (1.3–2.1)	0.79 (0.67–0.92)

PPV = positive predictive value, NPV = negative predictive value, LR = likelihood ratio

## Discussion

This observational study shows that performance of the ED-PEWS is variable in four heterogeneous acute care settings in LMICs, ranging from a moderate to good discriminative ability. Despite the variability in performance, the ED-PEWS mostly outperforms existing triage and the Egdell-PAWS. The predefined cut-off points of high and low urgency seem reasonable, but recalibration of the model by selecting the optimal cut-off values can improve performance for different settings. The most important predictors of high urgency in our study population are consciousness and work of breathing.

In the original study on the development of the ED-PEWS, cross-validation of the score in five European emergency departments resulted in an AUC of 0.86 (95%CI 0.84–0.88) for high urgency and 0.67 (95%CI 0.64–0.69) for high and intermediate urgency [6]. Because the initial study used a three-category reference standard, the performance in LMICs cannot be directly compared. What is most remarkable, however, are the large differences in performance between the LMIC settings themselves. Our study has not been designed to further evaluate the reasons behind the differences. However, it can be noted that the two settings that show the best performance, Gambia urban and Suriname are the settings with the highest level of resources, providing second- and third-line care. It is known that a prediction rule generally performs best in a population most similar to the original validation population [23, 24]. The outcome measure for the Suriname setting was also most comparable to the one from the original derivation study. The different inclusion criteria of the different cohorts do not seem to play a role in the differences in performance. Fever was an inclusion criterion for both cohorts from Gambia Urban and Tanzania, but performance in these settings was very different.

Although performance of the ED-PEWS is worst in the primary care settings in Tanzania and Gambia rural, its performance is still better than the original local triage system and the Egdell-PAWS for predicting high acuity in children. This might indicate that in these populations, scores solely based on vital parameters may be suboptimal to assess children’s urgency. This could be due to differences in the prevalence and severity of the presenting conditions. Alternatively, other factors may play a role in determining the urgency of a child, such as body composition or nutritional status [7, 23].

The most important predictors from the original study were consciousness and work of breathing, which is comparable to our findings in LMICs. However, although capillary refill time was a significant predictor in the original study, in the current analysis it was only significant in Suriname but not in Gambia rural and contributed little in Gambia urban. This could be due to differences in measurement, for example as a consequence of differences in skin colour or rates of anaemia, or could be related to the variability in underlying conditions.

### Strengths and limitations

To the best of our knowledge, the current study is one of the few evaluating a PEWS in LMICs in a multicentre study [25]. We were able to include large datasets from four diverse acute care settings.

Earlier research on the development and use of PEWS has mostly been performed in large tertiary care hospitals in LMICs [26–30]. Our study includes two databases with data from primary care settings, one from a rural area, which is unique for research in LMICs. Other studies on the validation of a PEWS in LMICs mostly focused on a single outcome measure, such as mortality, and used PEWS based on expert opinion. This study focuses on a combined outcome measure to better assess high urgency and uses a PEWS based on statistical analyses. Furthermore, the carefully collected clinical data and the low number of missing values across all settings are a strength of this study.

Our study also has some limitations. There is no gold standard that reflects patient urgency in acute care settings [31]. Therefore we used the reference standard that was applied in the original ED-PEWS validation and adapted this standard to the different settings [6]. In close collaboration with the local research teams and based on the available data, we developed an outcome measure that was the best possible reflection of patient urgency in these different centres. However, these outcome measures are not completely objective, indicating that different healthcare workers may make different decisions whether to refer, hospitalize or administer parenteral drugs for a child. These differences in outcome measure could explain the difference in high-urgent children in the settings together with the level of care and healthcare system in the country. In addition, for some items there is a risk of circularity, because vital signs influence treatment decisions. However, because the ED-PEWS consists of multiple items combined in a composite score, no vital sign alone is able to predict the outcome. Also, the cut-offs for the treatment decisions and the cut-offs used in the ED-PEWS are likely different.

The databases from Gambia urban and Tanzania only included children with fever. Therefore, we could not draw any conclusions about the general ED population in these settings. Also, the Tanzanian database did not include capillary refill time, which is a limitation of this data. Moreover, there was a limited number of outcomes in the cohort from Gambia Urban. Finally, due to the heterogeneity of the settings we were unable to pool the results and had to present the results of each setting separately.

Due to the fact that we used existing databases, we had no direct effect on the accuracy of measurements, however all of the original studies tried to minimalize this bias by assuring complete and accurate data.

Our study compares performance of the ED-PEWS with the locally used triage systems, amongst others the widely used ETAT, ESI, and IMCI guidelines. This comparison is solely based on the recognition of high urgency children, and it must be noted that these systems have a much broader use. For example, the WHO ETAT also functions as a mechanism to identify and start treatment of children with life threatening conditions, which we did not assess in our study.

### Future research

The majority of children in the world live in LMICs [32]. The implementation of PEWS in these countries may provide better identification of the sickest children and better allocation of resources, which could decrease mortality and morbidity. In health care settings in LMICs with a higher level of care, adding the ED-PEWS to the first assessment of children appears promising. However, additional external validation of the ED-PEWS is needed, preferably in an implementation study to assess its true clinical value. Implementing a novel PEWS in acute care settings would require training of healthcare workers and guidance on what to do based on a certain score. Furthermore, in clinical settings the ED-PEWS would become isolated from subsequent measures. In implementation it is important to look if this early warning score should be added to an existing triage system and which consequences should be assigned after the use of the ED-PEWS. In parallel to the performance of the ED-PEWS, this study also gives methodological insights in showing how data from such different sites can be combined in future prospective studies. The study clearly demonstrates that it is important to take in account the huge variability between settings in LMICs.

In addition, including more centres in the evaluation of the ED-PEWS could provide more insight in the performance of this early warning score in the various acute care settings. In primary care settings, optimising the ED-PEWS is needed to improve discrimination. In addition, further research should focus on novel predictors and other contextual variables influencing the predictive values of the vital parameters in these rural areas, such as access to health care and nutritional status. This further research could give us more insight in the differences between the performances of the various settings. Additionally, research on the influence of social and parental determinants on these factors could provide more understanding in the variability of these settings.

## Supporting information

S1 File Evidence before this study. (DOCX)

S2 File Definitions of physiological parameters and measurements in the different study sites. (DOCX)

S3 File Emergency Department-Paediatric Early Warning Score. (DOCX)

S4 File Reference standard of original validation study. (DOCX)

S5 File Paediatric Advanced Warning score. (DOCX)

S6 File Exclusion criteria of the databases. (DOCX)

S7 File Baseline characteristics physiological parameters. (DOCX)

S8 File Comparison validation and development data regarding the distribution of important variables. (DOCX)

S9 File Association between parameters of the ED-PEWS and high urgency. (DOCX)

S10 File Optimal cut-off points. (DOCX)

S11 File VITALS study group author list. (DOCX)
