# Supplementary material for: Validation of the Emergency Department-Paediatric Early Warning Score (ED-PEWS) for use in low- and middle-income countries: A multicentre observational study
Source: PLOS Glob Public Health. 2024 Mar 21;4(3):e0002716. doi: 10.1371/journal.pgph.0002716 (PMC10956749; doi:10.1371/journal.pgph.0002716)
Supplement: S1 File — (DOCX) [file pgph.0002716.s001.docx]

**S1 File. Evidence before this study**

We performed a literature search to identify evidence on the use of paediatric early warning scores (PEWS) in acute care settings in low- and middle income countries (LMICs). The search was conducted in PubMed from inception until March 15, 2023 using the search terms “PEWS”, “Early Warning Score”, “Severity of illness”, “Priority score” and “Illness detection” in combination with terms related to “child” and to “low- and middle-income countries”, including the names of every low- and middle-income country. Sixteen relevant articles described the implementation, validation or use of a PEWS in a LMIC, and nine different PEWS were used. Fifteen articles were conducted in tertiary care hospitals or large referral hospitals and only one article described the implementation of a PEWS in a rural district hospital. Three articles described the validity of a PEWS originally developed in a high-income country, four articles used adapted these PEWS to resource limited settings and nine articles used PEWS developed in LMICs. Most PEWS were based on expert opinion. One PEWS was developed based on statistical analysis but only assessed mortality as the outcome. Furthermore fifteen out of sixteen articles were performed in a single study site. There is a need for a PEWS that is robustly validated and applicable in emergency care facilities in LMICs to improve the identification of high urgency children in these settings.
