# Supplementary material for: Validation of the Emergency Department-Paediatric Early Warning Score (ED-PEWS) for use in low- and middle-income countries: A multicentre observational study
Source: PLOS Glob Public Health. 2024 Mar 21;4(3):e0002716. doi: 10.1371/journal.pgph.0002716 (PMC10956749; doi:10.1371/journal.pgph.0002716)
Supplement: S2 File — (DOCX) [file pgph.0002716.s002.docx]

**S2 File. Definitions of physiological parameters and measurements in the different study sites**

|  | **Definition** | **Gambia Rural** | **Gambia Urban** | **Suriname** | **Tanzania** |
| --- | --- | --- | --- | --- | --- |
| **Heart rate** | Beats per minute | By pulse oximetry | By pulse oximetry | By monitoring device | By pulse oximetry |
| **Respiratory rate** | Breaths per minute | By manual count | By manual count | By manual count | By manual count |
| **Oxygen saturation** | Percentage on room air | By pulse oximetry | By pulse oximetry | By pulse oximetry | By pulse oximetry |
| **Consciousness** | Normal: Alert (AVPU), GCS 15, BCS 5, or clinically observed  Abnormal: any other | As measured by GCS | As measured by BCS | As measured by AVPU | Defined by clinical observation |
| **Capillary refill time** | Normal: < 3 seconds  Abnormal: ≥ 3 seconds | Centrally measured by pressing on skin for 5 seconds | Centrally measured by pressing on skin for 5 seconds | Centrally measured by pressing on skin for 5 seconds | NA |
| **Work of breathing** | Normal: no abnormalities  Abnormal: presence of respiratory distress | Several yes/no items in patient’s record | Work of breathing as item in patient’s record | Work of breathing as item in patient’s record | Work of breathing as item in patient’s record |
| **Temperature** | Degrees Celsius | Axillary thermometer | Axillary thermometer | Ear thermometer or forehead thermometer | Axillary thermometer |
