# Supplementary figures and images for: Validation of the Emergency Department-Paediatric Early Warning Score (ED-PEWS) for use in low- and middle-income countries: A multicentre observational study
Source: PLOS Glob Public Health. 2024 Mar 21;4(3):e0002716. doi: 10.1371/journal.pgph.0002716 (PMC10956749; doi:10.1371/journal.pgph.0002716)

**S3 File. Emergency Department-Paediatric Early Warning Score**

**
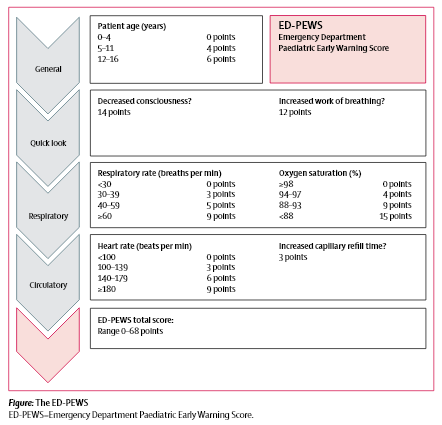
**

Supplement: S3 File — (DOCX) [file pgph.0002716.s003.docx]

**S5 File. Paediatric Advanced Warning score**


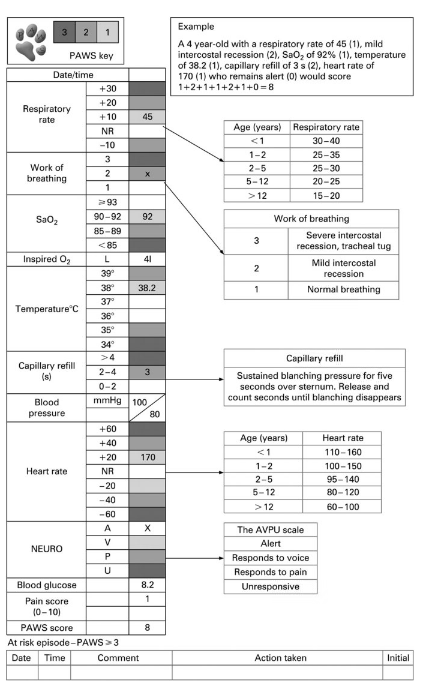

Supplement: S5 File — (DOCX) [file pgph.0002716.s005.docx]
