# Supplementary material for: Validation of the Emergency Department-Paediatric Early Warning Score (ED-PEWS) for use in low- and middle-income countries: A multicentre observational study
Source: PLOS Glob Public Health. 2024 Mar 21;4(3):e0002716. doi: 10.1371/journal.pgph.0002716 (PMC10956749; doi:10.1371/journal.pgph.0002716)
Supplement: S4 File — (DOCX) [file pgph.0002716.s004.docx]

**S4 File. Reference standard of original validation study**

3-category reference standard as proxy for true patient urgency^1^

| **Urgency category** | **Items** | **Time before being seen by a physician** |
| --- | --- | --- |
| High urgency | - Mortality at the ED, *and/or* - ICU admission immediately after the ED visit, *and/or* - Immediate lifesaving interventions*, *and/or* - Oxygen administration (indicating hypoxemia or serious illness requiring advanced paediatric life support guidelines | 10 minutes |
| Intermediate urgency | - Hospital admission immediately after the ED visit, *and/or* - IV medication or fluids or inhalation medication at the ED, *and/or* - >1 of the following: Radiology; Lab test; Oral medication | < 60 minutes |
| Low urgency | - None of the above | ≥ 60 minutes |

* Immediate lifesaving interventions are defined as any of the following^2,3^:
 - airway/breathing support (e.g. intubation or emergent noninvasive positive pressure ventilation);
 - electrical therapy (e.g. defibrillation, emergent cardioversion or external pacing);
 - emergency procedures (e.g. chest needle decompression, pericardiocentesis, or open
 thoracotomy)
 - haemodynamic support (e.g. significant IV fluid in case of hypotension, blood administration or
 control of major bleeding) or emergency medications (e.g. atropine, adenosine, inotropics,
 epinephrine, nalaxon, dextrose in case of hypoglycaemia)

**References:**

1. Zachariasse JM, Nieboer D, Maconochie IK, et al. Development and validation of a Paediatric Early Warning Score for use in the emergency department. Lancet Child Adolesc Health. 2020 Aug;4(8):583-591.
2. Lee JY, Oh SH, Peck EH, et al. The validity of the Canadian Triage and Acuity Scale in predicting resource utilization and the need for immediate life-saving interventions in elderly emergency department patients. Scand J Trauma Resusc Emerg Med. 2011;19:68.
3. Platts-Mills TF, Travers D, Biese K, et al. Accuracy of the Emergency Severity Index triage instrument for identifying elder emergency department patients receiving an immediate life-saving intervention. Acad Emerg Med· 2010;17(3):238-243·
