# Supplementary material for: Validation of the Emergency Department-Paediatric Early Warning Score (ED-PEWS) for use in low- and middle-income countries: A multicentre observational study
Source: PLOS Glob Public Health. 2024 Mar 21;4(3):e0002716. doi: 10.1371/journal.pgph.0002716 (PMC10956749; doi:10.1371/journal.pgph.0002716)
Supplement: S6 File — (DOCX) [file pgph.0002716.s006.docx]

**S6 File. Exclusion criteria of the databases**

**Gambia Rural original database**

Original exclusion criteria:

- Duplicates

- Children with missing data on age

- Children with mothers under fifteen years of age

Total: 49,346 visits

Total: 41,917 visits

Excluded:

- 7,429 non-acute visits

Figure 6.1 Exclusion criteria of the Gambia Rural database

**Gambia Urban original database**

Original inclusion criteria:

- Children < 18 years of age with fever and an indication for a blood draw

Total: 501

Total: 501 visits

Excluded:

- None

Figure 6.2 Exclusion criteria of the Gambia Urban database

**Suriname original database**

Original exclusion criteria:

- None

Total: 2,638

Total: 2,608 visits

Excluded:

- 30 children with age > 16 years

Figure 6.3 Exclusion criteria of the Suriname database

**Tanzania original database**

Original exclusion criteria:

- Weight less than 2.5kg

- Main complaint being injury/acute poisoning

- Previous medical care for present illness

Total: 3,192

Total: 1,596 visits

Excluded:

- 1,596 from the ALMANACH group

Figure 6.4 Exclusion criteria of the Tanzania database
