# Supplementary material for: Validation of the Emergency Department-Paediatric Early Warning Score (ED-PEWS) for use in low- and middle-income countries: A multicentre observational study
Source: PLOS Glob Public Health. 2024 Mar 21;4(3):e0002716. doi: 10.1371/journal.pgph.0002716 (PMC10956749; doi:10.1371/journal.pgph.0002716)
Supplement: S7 File — (DOCX) [file pgph.0002716.s007.docx]

**S7 File. Baseline characteristics physiological parameters**

|  | **Gambia Rural**  **(n=41 917)** | **Gambia Urban**  **(n=501)** | **Suriname**  **(n= 2 608)** | **Tanzania**  **(n=1 596)** |
| --- | --- | --- | --- | --- |
| Heart rate, n (%) | | | | |
| 0 points | 14 182 (33.8) | 11 (2.2) | 656 (25.2) | 2 (0.2) |
| 3 points | 20 754 (49.5) | 198 (39.5) | 1 332 (51.1) | 585 (36.7) |
| 6 points | 6 643 (15.8) | 253 (50.5) | 508 (19.5) | 976 (61.2) |
| 9 points | 234 (0.6) | 39 (7.8) | 59 (2.3) | 31 (1.9) |
| Unknown | 113 (0.3) | 0 (0) | 53 (2.0) | 2 (0.1) |
| Respiratory Rate, n (%) |  |  |  |  |
| 0 points | 26 047 (62.2) | 67 (13.4) | 1 997 (75.8) | 102 (6.4) |
| 3 points | 13 944 (33.3) | 157 (31.3) | 272 (10.4) | 547 (34.3) |
| 5 points | 1 392 (3.3) | 222 (44.3) | 194 (7.4) | 849 (53.2) |
| 9 points | 277 (0.7) | 55 (11.0) | 40 (1.5) | 95 (6.0) |
| Unknown | 257 (0.6) | 0 (0) | 125 (4.8) | 3 (0.2) |
| Oxygen Saturation, n (%) | |  |  |  |
| 0 points | 37 441 (89.3) | 338 (67.5) | 1 756 (67.4) | 1 014 (63.5) |
| 4 points | 2 342 (5.6) | 155 (30.9) | 666 (25.5) | 541 (33.9) |
| 9 points | 107 (0.3) | 7 (1.4) | 106 (4.1) | 34 (2.1) |
| 15 points | 33 (0.1) | 1 (0.2) | 27 (1.0) | 4 (0.3) |
| Unknown | 1994 (4.8) | 0 (0) | 53 (2.0) | 3 (0.2) |
| Capillary Refill Time, n (%) | | | | |
| 0 points | 40 987 (97.8) | 501 (100.0) | 2 496 (95.7) | - |
| 3 points | 213 (0.5) | 0 (0) | 56 (2.1) | - |
| Unknown | 717 (1.7) | 0 (0) | 56 (2.1) | - |
| Consciousness, n (%) | | | | |
| 0 points | 40 890 (97.5) | 500 (99.8) | 2 495 (95.7) | 1 587 (99.4) |
| 14 points | 439 (1.3) | 1 (0.2) | 92 (3.5) | 9 (0.6) |
| Unknown | 498 (1.2) | 0 (0.0) | 21 (0.8) | - |
| Increased work of breathing, n (%) | | | | |
| 0 points | 41 323 (98.6) | 461 (92.0) | 2 264 (86.8) | 724 (45.4) |
| 12 points | 544 (1.3) | 39 (7.8) | 274 (10.5) | 164 (10.3) |
| Unknown | 50 (0.1) | 1 (0.2) | 70 (2.7) | 708 (44.4) |
