# Supplementary material for: Validation of the Emergency Department-Paediatric Early Warning Score (ED-PEWS) for use in low- and middle-income countries: A multicentre observational study
Source: PLOS Glob Public Health. 2024 Mar 21;4(3):e0002716. doi: 10.1371/journal.pgph.0002716 (PMC10956749; doi:10.1371/journal.pgph.0002716)
Supplement: S8 File — (DOCX) [file pgph.0002716.s008.docx]

**S8 File. Comparison validation and development data regarding the distribution of important variables**

|  | **Validation** | | | | **Development** | | | | |
| --- | --- | --- | --- | --- | --- | --- | --- | --- | --- |
|  | **Gambia Rural**  **(n=41 917)** | **Gambia Urban**  **(n=501)** | **Suriname**  **(n= 2 608)** | **Tanzania**  **(n=1 596)** | **Erasmus MC**  **(n=18,594)** | **Maasstad Hospital**  **(n=10,584)** | **St Mary’s Hospital**  **(n=15,556)** | **Hospital Fernando da Fonseca**  **(n=53,175)** | **General Hospital, Vienna**  **(n=21,300)** |
| **Demographics** | | | | | | | | | |
| Age | | | | | | | | | |
| Median (IQR) | 4.2 (1.5-9.7) | 3.1 (1.6-5.4) | 4.0 (1.7-8.8) | 1.1 (0.7-1.9)* | 4.3 (1.4-9.8) | 5.7 (1.9-11.6) | 3.9 (1.5-8.8) | 4.7 (1.9-9.5) | 3.9 (1.6-8.3) |
| Sex, n (%) | | | | | | | | | |
| Male | 21,649 (52.6) | 264 (52.7) | 1,479 (56.7) | 891 (55.8) | 10,774 (57.9) | 6,004 (56.7) | 8,677 (55.8) | 27,685 (52.1) | 11,233 (52.7) |
| Female | 20,268 (48.4) | 237 (47.3) | 1,126 (43.2) | 705 (44.2) | 7,820 (42.1) | 4,580 (43.3) | 6,879 (44.2) | 25,490 (47.9) | 10,067 (47.3) |
| Unknown | - | - | 3 (0.1) | - | - | - | - | - | - |
| **Predictors** | | | | | | | | | |
| Heart rate, n (%) | | | | | | | | | |
| 0 points | 14 182 (33.8) | 11 (2.2) | 656 (25.2) | 2 (0.2) | 11,566 (62.2) | 7,387 (0.70) | 6,259 (40.2) | 28,404 (53.4) | 15,669 (73.6) |
| 3 points | 20 754 (49.5) | 198 (39.5) | 1 332 (51.1) | 585 (36.7) | 4,211 (22.6) | 1,495 (14.1) | 6,212 (39.9) | 17,892 (33.6) | 4,151 (19.5) |
| 6 points | 6 643 (15.8) | 253 (50.5) | 508 (19.5) | 976 (61.2) | 2,439 (13.1) | 1,411 (13.3) | 2,888 (18.6) | 6,395 (12.0) | 1,377 (6.5) |
| 9 points | 234 (0.6) | 39 (7.8) | 59 (2.3) | 31 (1.9) | 378 (2.0) | 291 (2.7) | 197 (1.3) | 484 (0.9) | 103 (0.5) |
| Unknown | 113 (0.3) | 0 (0) | 53 (2.0) | 2 (0.1) | - | - | - | - | - |
| Respiratory Rate, n (%) | | | | | | | | | |
| 0 points | 26 047 (62.2) | 67 (13.4) | 1 997 (75.8) | 102 (6.4) | 15,628 (84.0) | 9,384 (88.7) | 11,190 (71.9) | 43,584 (82.0) | 20,553 (96.5) |
| 3 points | 13 944 (33.3) | 157 (31.3) | 272 (10.4) | 547 (34.3) | 1,458 (7.8) | 396 (3.7) | 2,528 (16.3) | 6,890 (13.0) | 457 (2.1) |
| 5 points | 1 392 (3.3) | 222 (44.3) | 194 (7.4) | 849 (53.2) | 1,196 (6.4) | 556 (5.3) | 1,662 (10.7) | 2,435 (4.6) | 250 (1.2) |
| 9 points | 277 (0.7) | 55 (11.0) | 40 (1.5) | 95 (6.0) | 312 (1.7) | 248 (2.3) | 176 (1.1) | 266 (0.5) | 40 (0.2) |
| Unknown | 257 (0.6) | 0 (0) | 125 (4.8) | 3 (0.2) | - | - | - | - | - |
| Oxygen Saturation, n (%) | | | | | | | | | |
| 0 points | 37 441 (89.3) | 338 (67.5) | 1 756 (67.4) | 1 014 (63.5) | 16,910 (90.9) | 9,417 (89.0) | 13,524 (86.9) | 43,166 (81.2) | 19,455 (91.3) |
| 4 points | 2 342 (5.6) | 155 (30.9) | 666 (25.5) | 541 (33.9) | 1254 (6.7) | 990 (9.4) | 1,848 (11.9) | 9,348 (17.6) | 1,651 (7.8) |
| 9 points | 107 (0.3) | 7 (1.4) | 106 (4.1) | 34 (2.1) | 279 (1.5) | 156 (1.5) | 160 (1.0) | 627 (1.2) | 152 (0.7) |
| 15 points | 33 (0.1) | 1 (0.2) | 27 (1.0) | 4 (0.3) | 151 (0.1) | 21 (0.2) | 24 (0.2) | 34 (0.1) | 42 (0.2) |
| Unknown | 1994 (4.8) | 0 (0) | 53 (2.0) | 3 (0.2) | - | - | - | - | - |
| Capillary Refill Time, n (%) | | | | | | | | | |
| 0 points | 40 987 (97.8) | 501 (100.0) | 2 496 (95.7) | - | 18,464 (99.3) | 10,376 (98.0) | 15,446 (99.3) | 52.881 (99.4) | 21,279 (99.9) |
| 3 points | 213 (0.5) | 0 (0) | 56 (2.1) | - | 130 (0.7) | 208 (2.0) | 110 (0.7) | 294 (0.6) | 21 (0.1) |
| Unknown | 717 (1.7) | 0 (0) | 56 (2.1) | - | - | - | - | - | - |
| Consciousness, n (%) | | | | | | | | | |
| 0 points | 40 890 (97.5) | 500 (99.8) | 2 495 (95.7) | 1 587 (99.4) | 17,784 (95.6) | 10,488 (99.1) | 15,504 (99.7) | 53,099 (99.9) | 21,225 (99.6) |
| 14 points | 439 (1.3) | 1 (0.2) | 92 (3.5) | 9 (0.6) | 810 (4.4) | 96 (0.9) | 52 (0.3) | 76 (0.1) | 75 (0.4) |
| Unknown | 498 (1.2) | 0 (0.0) | 21 (0.8) | - | - | - | - | - | - |
| Increased work of breathing, n (%) | | | | | | | | | |
| 0 points | 41 323 (98.6) | 461 (92.0) | 2 264 (86.8) | 724 (45.4) | 17,288 (93.0) | 9,693 (91.6) | 14,333 (92.1) | 48,375 (91.0) | 20,532 (96.4) |
| 12 points | 544 (1.3) | 39 (7.8) | 274 (10.5) | 164 (10.3) | 1,306 (7.0) | 891 (8.4) | 1,223 (7.9) | 4,800 (9.0) | 768 (3.6) |
| Unknown | 50 (0.1) | 1 (0.2) | 70 (2.7) | 708 (44.4) | - | - | - | - | - |
| **Outcome** | | | | | | | | | |
| Outcome measure, n (%) | | | | | | | | | |
| 2-level: High urgent | 2,054 (4.9) | 23 (4.6) | 648 (24.8) | 106 (6.6) | - | - | - | - | - |
| 2-level: Not high urgent | 39,863 (95.1) | 478 (95.4) | 1,960 (75.2) | 1,490 (93.4) | - | - | - | - | - |
| 3-level: High urgent | - | - | - | - | 1,335 (7.2) | 129 (1.2) | 275 (1.8) | 141 (0.3) | 127 (0.6) |
| 3-level: Intermediate urgent | - | - | - | - | 5,940 (31.9) | 4,193 (39.6) | 3,211 (20.6) | 11,571 (21.8) | 4,212 (19.8) |
| 3-level: Low urgent | - | - | - | - | 11,319 (60.0) | 6,262 (59.2) | 12,070 (77.6) | 41,463 (78.0) | 16,961 (79.6) |

* Inclusion criteria include age
