# Supplementary material for: Validation of the Emergency Department-Paediatric Early Warning Score (ED-PEWS) for use in low- and middle-income countries: A multicentre observational study
Source: PLOS Glob Public Health. 2024 Mar 21;4(3):e0002716. doi: 10.1371/journal.pgph.0002716 (PMC10956749; doi:10.1371/journal.pgph.0002716)
Supplement: S9 File — (DOCX) [file pgph.0002716.s009.docx]

**S9 File. Association between parameters of the ED-PEWS and high urgency**

|  | **Gambia Rural** | | **Gambia Urban** | | **Suriname** | | **Tanzania** | |
| --- | --- | --- | --- | --- | --- | --- | --- | --- |
|  | **OR (95%CI)** | **aOR (95%CI)** | **OR (95%CI)** | **aOR (95%CI)** | **OR (95%CI)** | **aOR (95%CI)** | **OR (95%CI)** | **aOR (95%CI)** |
| Age (years) |  |  |  |  |  |  |  |  |
| < 1 | Reference | Reference | Reference | Reference | Reference | Reference | Reference | Reference |
| 1 to <2 | 0.8 (0.7-0.9)* | 1.3 (1.1-1.5)* | 0.2 (0.0-0.7)* | 0.1 (0.0-0.9)* | 0.9 (0.7-1.3) | 1.2 (0.8-1.9) | 0.9 (0.6-1.4) | 1.1 (0.7-1.8) |
| 2 to <5 | 0.6 (0.6-0.7)* | 1.4 (1.2-1.6)* | 0.1 (0.0-0.5)* | 0.3 (0.1-1.6) | 0.8 (0.6-1.1) | 1.3 (0.9-2.0) | 1.1 (0.7-1.8) | 1.6 (0.9-2.8) |
| 5 to <12 | 0.4 (0.3-0.4)* | 1.6 (1.3-2.0)* | 0.4 (0.1-1.1) | 1.9 (0.4-10.5) | 0.7 (0.5-0.9)* | 2.1 (1.3-3.3)* | ·· | ·· |
| >12 | 0.3 (0.3-0.4)* | 2.1 (1.6-2.7)* | 0.7 (0.1-6.4) | 3.8 (0.2-64.9) | 1.1 (0.8-1.5) | 4.2 (2.5-7.1)* | ·· | ·· |
| Heart rate (beats per minute) | 1.0 (1.0-1.0)* | 1.0 (1.0-1.0)* | 1.0 (1.0-1.0)* | 1.0 (1.0-1.0)* | 1.0 (1.0-1.0)* | 1.0 (1.0-1.0)* | 1.0 (1.0-1.0)* | 1.0 (1.0-1.0)* |
| Respiratory Rate (breaths per minute) | 1.1 (1.1-1.1)* | 1.1 (1.1-1.1)* | 1.1 (1.1-1.1)* | 1.1 (1.0-1.1)* | 1.1 (1.1-1.1)* | 1.1 (1.0-1.1)* | 1.0 (1.0-1.1)* | 1.0 (1.0-1.1)* |
| 100- Oxygen Saturation | 1.1 (1.1-1.2)* | 1.0 (1.0-1.1)* | 1.4 (1.1-1.6)* | 1.3 (1.0-1.6)* | 1.5 (1.4-1.5)* | 1.2 (1.1-1.3)* | 1.1 (1.0-1.2)* | 1.1 (1.0-1.2)* |
| Capillary Refill Time |  |  |  |  |  |  |  |  |
| Abnormal | 1.0 (0.5-1.8) | 1.1 (0.6-2.2) | NA^a^ | NA^a^ | 4.2 (2.4-7.2)* | 3.5 (1.8-7.0)* | NA | NA |
| Consciousness |  |  |  |  |  |  |  |  |
| Decreased | 1.7 (1.2-2.3)* | 1.8 (1.3-2.5)* | NA^a^ | NA^a^ | 5.8 (3.8-9.0)* | 5.5 (3.3-9.3)* | NA^a^ | NA^a^ |
| Work of breathing |  |  |  |  |  |  |  |  |
| Increased work of breathing | 10.0 (8.3-12.1)* | 2.6 (2.0-3.3)* | 9.6 (3.8-24.0)* | 7.3 (2.2-24.2)* | 24.0 (17.1-33.6)* | 9.6 (6.4-14.3)* | 1.7 (1.0-3.0)* | 1.5 (0.8-2.7) |

OR = odds ratio, aOR = adjusted odds ratio

* p-value <0.05

^a^ Variable was available but with insufficient abnormal values to include in the analysis
