# Supplementary material for: Validation of the Emergency Department-Paediatric Early Warning Score (ED-PEWS) for use in low- and middle-income countries: A multicentre observational study
Source: PLOS Glob Public Health. 2024 Mar 21;4(3):e0002716. doi: 10.1371/journal.pgph.0002716 (PMC10956749; doi:10.1371/journal.pgph.0002716)
Supplement: S10 File — (DOCX) [file pgph.0002716.s010.docx]

**S10 File. Optimal cut-off points**

**Table 10.1 Diagnostic accuracy of the Emergency Department-Paediatric Early Warning score low-urgency cut-offs**

| **Cut-off** | **Patients classified (%)** | **Sensitivity (95%CI)** | **Specificity (95%CI)** | **NPV (95%CI)** | **PPV (95%CI)** | **Positive LR (95%CI)** | **Negative LR (95%CI)** |
| --- | --- | --- | --- | --- | --- | --- | --- |
| **Gambia Rural** | | |  |  |  |  |  |
| <5 | 33.0 | 0.83 (0.81-0.84) | 0.34 (0.33-0.34) | 0.97 (0.97-0.98) | 0.06 (0.06-0.06) | 1.3 (1.2-1.3) | 0.51 (0.46-0.56) |
| <6 | 33.0 | 0.83 (0.81-0.84) | 0.34 (0.33-0.34) | 0.97 (0.97-0.98) | 0.06 (0.06-0.06) | 1.3 (1.2-1.3) | 0.51 (0.46-0.56) |
| <7 | 65.9 | 0.57 (0.55-0.59) | 0.67 (0.67-0.67) | 0.97 (0.97-0.97) | 0.08 (0.08-0.08) | 1.7 (1.7-1.8) | 0.65 (0.61-0.68) |
| <8 | 77.3 | 0.49 (0.47-0.51) | 0.79 (0.78-0.79) | 0.97 (0.97-0.97) | 0.11 (0.10-0.11) | 2.3 (2.2-2.4) | 0.65 (0.62-0.68) |
| <9 | 78.6 | 0.46 (0.44-0.49) | 0.80 (0.79-0.80) | 0.97 (0.97-0.97) | 0.11 (0.10-0.11) | 2.3 (2.2-2.4) | 0.67 (0.64-0.70) |
| <10 | 89.9 | 0.30 (0.29-0.33) | 0.91 (0.91-0.91) | 0.96 (0.96-0.96) | 0.14 (0.14-0.16) | 3.4 (3.1-3.6) | 0.76 (0.74-0.79) |
| **Gambia Urban** | |  |  |  |  |  |  |
| <5 | 4.8 | 1.00 (0.85-1.00) | 0.05 (0.03-0.07) | 1.00 (1.00-1.00) | 0.05 (0.05-0.05) | 1.05 (1.03-1.07) | 0.00 (0.00-0.00) |
| <6 | 5.4 | 1.00 (0.85-1.00) | 0.06 (0.04-0.08) | 1.00 (1.00-1.00) | 0.05 (0.05-0.05) | 1.06 (1.04-1.08) | 0.00 (0.00-0.00) |
| <7 | 10.8 | 1.00 (0.85-1.00) | 0.11 (0.08-0.14) | 1.00 (1.00-1.00) | 0.05 (0.05-0.05) | 1.13 (1.09-1.16) | 0.00 (0.00-0.00) |
| <8 | 15.2 | 1.00 (0.85-1.00) | 0.15 (0.13-0.19) | 1.00 (1.00-1.00) | 0.05 (0.05-0.05) | 1.19 (1.14-1.24) | 0.00 (0.00-0.00) |
| <9 | 19.8 | 0.96 (0.78-1.00) | 0.21 (0.17-0.24) | 0.99 (0.93-1.00) | 0.05 (0.05-0.06) | 1.20 (1.09-1.33) | 0.21 (0.03-1.45) |
| <10 | 28.5 | 0.96 (0.78-1.00) | 0.30 (0.26-0.34) | 0.99 (0.95-1.00) | 0.06 (0.06-0.07) | 1.36 (1.23-1.51) | 0.15 (0.02-1.00) |
| **Suriname** | | |  |  |  |  |  |
| <5 | 32.3 | 0.87 (0.85-0.90) | 0.39 (0.37-0.41) | 0.90 (0.88-0.92) | 0.32 (0.31-0.33) | 1.4 (1.4-1.5) | 0.33 (0.26-0.40) |
| <6 | 32.5 | 0.87 (0.84-0.89) | 0.39 (0.37-0.41) | 0.90 (0.88-0.92) | 0.32 (0.31-0.33) | 1.4 (1.4-1.5) | 0.33 (0.27-0.41) |
| <7 | 45.4 | 0.79 (0.76-0.82) | 0.53 (0.51-0.56) | 0.89 (0.87-0.90) | 0.36 (0.35-0.37) | 1.7 (1.6-1.8) | 0.39 (0.34-0.46) |
| <8 | 61.7 | 0.71 (0.67-0.75) | 0.73 (0.71-0.75) | 0.88 (0.87-0.90) | 0.46 (0.44-0.48) | 2.6 (2.4-2.8) | 0.40 (0.35-0.45) |
| <9 | 63.9 | 0.69 (0.65-0.73) | 0.75 (0.73-0.77) | 0.88 (0.87-0.89) | 0.47 (0.45-0.50) | 2.7 (2.5-3.0) | 0.41 (0.37-0.47) |
| <10 | 68.7 | 0.65 (0.61-0.68) | 0.80 (0.78-0.82) | 0.87 (0.86-0.88) | 0.51 (0.49-0.54) | 3.2 (2.9-3.5) | 0.44 (0.40-0.49) |

**Table 10.2 Diagnostic accuracy of the Emergency Department-Paediatric Early Warning Score high-urgency cut-off points**

| **Cut-off** | **Patients classified (%)** | **Sensitivity (95%CI)** | **Specificity (95%CI)** | **NPV (95%CI)** | **PPV (95%CI)** | **Positive LR (95%CI)** | **Negative LR (95%CI)** |
| --- | --- | --- | --- | --- | --- | --- | --- |
| **Gambia Rural** | | |  |  |  |  |  |
| ≥30 | 0.2 | 0.03 (0.02-0.04) | 1.00 (1.00-1.00) | 0.95 (0.95-0.95) | 0.64 (0.54-0.74) | 35.2 (22.9-54.0) | 0.97 (0.97-0.98) |
| ≥25 | 0.6 | 0.06 (0.05-0.07) | 1.00 (1.00-1.00) | 0.95 (0.95-0.95) | 0.53 (0.47-0.59) | 22.1 (17.1-28.5) | 0.94 (0.93-0.95) |
| ≥20 | 1.7 | 0.09 (0.08-0.11) | 0.99 (0.99-0.99) | 0.96 (0.95-0.96) | 0.26 (0.23-0.30) | 7.0 (5.9-8.2) | 0.92 (0.91-0.93) |
| ≥15 | 3.4 | 0.16 (0.15-0.18) | 0.97 (0.97-0.97) | 0.96 (0.96-0.96) | 0.23 (0.21-0.26) | 6.0 (5.3-6.7) | 0.86 (0.84-0.88) |
| ≥10 | 10.1 | 0.30 (0.29-0.33) | 0.91 (0.91-0.91) | 0.96 (0.96-0.96) | 0.14 (0.14-0.16) | 3.4 (3.1-3.6) | 0.76 (0.74-0.79) |
| **Gambia Urban** | |  |  |  |  |  |  |
| ≥30 | 2.4 | 0.17 (0.05-0.39) | 0.98 (0.97-0.99) | 0.96 (0.95-0.97) | 0.33 (0.14-0.61) | 10.39 (3.37-32.01) | 0.84 (0.70-1.01) |
| ≥25 | 5.6 | 0.30 (0.13-0.53) | 0.96 (0.93-0.97) | 0.97 (0.96-0.97) | 0.25 (0.14-0.41) | 6.93 (3.29-14.61) | 0.73 (0.55-0.95) |
| ≥20 | 8.4 | 0.39 (0.19-0.61) | 0.93 (0.91-0.95) | 0.97 (0.96-0.98) | 0.21 (0.13-0.33) | 5.83 (3.18-10.70) | 0.65 (0.47-0.91) |
| ≥15 | 30.9 | 0.65 (0.43-0.84) | 0.71 (0.66-0.75) | 0.98 (0.96-0.99) | 0.10 (0.07-0.13) | 2.23 (1.60-3.10) | 0.49 (0.28-0.86) |
| ≥10 | 71.5 | 0.96 (0.78-1.00) | 0.30 (0.26-0.34) | 0.99 (0.95-1.00) | 0.06 (0.06-0.07) | 1.36 (1.23-1.51) | 0.15 (0.02-1.00) |
| **Suriname** | | |  |  |  |  |  |
| ≥30 | 4.0 | 0.15 (0.12-0.18) | 1.00 (0.99-1.00) | 0.78 (0.77-0.79) | 0.94 (0.88-0.97) | 49.4 (21.8-112.1) | 0.85 (0.82-0.88) |
| ≥25 | 6.7 | 0.25 (0.22-0.29) | 0.99 (0.99-1.00) | 0.80 (0.79-0.81) | 0.93 (0.88-0.96) | 41.0 (22.9-73.0) | 0.75 (0.72-0.79) |
| ≥20 | 11.0 | 0.37 (0.33-0.41) | 0.98 (0.97-0.98) | 0.84 (0.79-0.87) | 0.82 (0.82-0.83) | 15.5 (11.5-21.0) | 0.64 (0.61-0.68) |
| ≥15 | 15.7 | 0.47 (0.43-0.51) | 0.95 (0.94-0.96) | 0.84 (0.84-0.85) | 0.75 (0.71-0.79) | 9.1 (7.4-11.2) | 0.56 (0.52-0.60) |
| ≥10 | 31.3 | 0.65 (0.61-0.68) | 0.80 (0.78-0.82) | 0.87 (0.86-0.88) | 0.51 (0.49-0.54) | 3.2 (2.9-3.5) | 0.44 (0.40-0.49) |
