# Supplementary material for: Validation of the Emergency Department-Paediatric Early Warning Score (ED-PEWS) for use in low- and middle-income countries: A multicentre observational study
Source: PLOS Glob Public Health. 2024 Mar 21;4(3):e0002716. doi: 10.1371/journal.pgph.0002716 (PMC10956749; doi:10.1371/journal.pgph.0002716)
Supplement: S11 File — (DOCX) [file pgph.0002716.s011.docx]

**S11 File. VITALS study group author list**

The VITaLs (VItal signs and Triage in Low- and middle income countries) study group includes the following:

Navin P. Boeddha
Department of General Paediatrics, Erasmus MC- Sophia Children’s Hospital, Rotterdam, The Netherlands

Natanael Holband
Department of Paediatrics, Academic Hospital Paramaribo, Paramaribo, Suriname

Abdoulie Faal
Applications Development & e-Health department, Medical Research Council Unit The Gambia at the London School of Hygiene and Tropical Medicine, Fajara, The Gambia

Amadu E. Juliana
Department of Paediatrics, Academic Hospital Paramaribo, Paramaribo, Suriname

Godfrey A. Kavishe
National Institute of Medical Research – Mbeya Medical Research Centre, Tanzania

Kristina Keitel
Division of Paediatric Emergency Medicine, Department of Paediatrics, Inselspital, Bern University Hospital, Bern, Switzerland
Swiss Tropical and Public Health Institute (SwissTPH), University of Basel, Switzerland

Naomi Kemps
Department of General Paediatrics, Erasmus MC- Sophia Children’s Hospital, University Medical Centre Rotterdam, Rotterdam, The Netherlands

Kevin H. van ’t Kruys
Department of Paediatrics, Academic Hospital Paramaribo, Paramaribo, Suriname

Elizabeth V. Ledger
Department of Paediatrics, Bristol Royal Hospital for Children, Bristol, United Kingdom

Henry Mark
Independent Consultant, Nottingham, United Kingdom

Henriëtte A. Moll
Department of General Paediatrics, Erasmus MC- Sophia Children’s Hospital, University Medical Centre Rotterdam, Rotterdam, The Netherlands

Andrew M. Prentice
Nutrition and Planetary Health Theme, Medical Research Council Unit The Gambia at the London School of Hygiene & Tropical Medicine, Banjul, The Gambia

Fatou Secka
Medical Research Council Unit The Gambia at the London School of Hygiene and Tropical Medicine, Fajara, The Gambia

Rainer Tan
Swiss Tropical and Public Health Institute (SwissTPH), University of Basel, Switzerland
Center for Primary Care and Public Health (Unisanté), University of Lausanne, Lausanne, Switzerland

Stefan A. Unger
Department of Child Life and Health, University of Edinburgh, Edinburgh, United Kingdom

Effua Usuf
Medical Research Council Unit The Gambia at the London School of Hygiene and Tropical Medicine, Fajara, The Gambia

Joany M. Zachariasse
Department of General Paediatrics, Erasmus MC- Sophia Children’s Hospital, University Medical Centre Rotterdam, Rotterdam, The Netherlands
